# Recent Insights into the HIV/AIDS Pandemic

**DOI:** 10.15698/mic2016.09.529

**Published:** 2016-09-05

**Authors:** Juan C. Becerra, Lukas S. Bildstein, Johannes S. Gach

**Affiliations:** 1 Department of Medicine, Division of Infectious Diseases, University of California, Irvine, Irvine, CA 92697, USA.; 2 Graphic Design, 6850 Dornbirn, Austria.

**Keywords:** HIV-1, AIDS, antiretroviral therapy, epidemiology, pathology, treatment, virus entry

## Abstract

Etiology, transmission and protection: Transmission of HIV, the causative agent of AIDS, occurs predominantly through bodily fluids. Factors that significantly alter the risk of HIV transmission include male circumcision, condom use, high viral load, and the presence of other sexually transmitted diseases. Pathology/Symptomatology: HIV infects preferentially CD4^+^ T lymphocytes, and Monocytes. Because of their central role in regulating the immune response, depletion of CD4^+^ T cells renders the infected individual incapable of adequately responding to microorganisms otherwise inconsequential. Epidemiology, incidence and prevalence: New HIV infections affect predominantly young heterosexual women and homosexual men. While the mortality rates of AIDS related causes have decreased globally in recent years due to the use of highly active antiretroviral therapy (HAART) treatment, a vaccine remains an elusive goal. Treatment and curability: For those afflicted HIV infection remains a serious illness. Nonetheless, the use of advanced therapeutics have transformed a dire scenario into a chronic condition with near average life spans. When to apply those remedies appears to be as important as the remedies themselves. The high rate of HIV replication and the ability to generate variants are central to the viral survival strategy and major barriers to be overcome. Molecular mechanisms of infection: In this review, we assemble new details on the molecular events from the attachment of the virus, to the assembly and release of the viral progeny. Yet, much remains to be learned as understanding of the molecular mechanisms used in viral replication and the measures engaged in the evasion of immune surveillance will be important to develop effective interventions to address the global HIV pandemic.

## EPIDEMIOLOGY, INCIDENCE AND PREVALENCE

Acquired immunodeficiency syndrome (AIDS), caused by chronic infection with the human immunodeficiency virus-1 (HIV-1), is one of the most devastating pandemics ever recorded in human history 1. Shortly after the first reports of AIDS in the United States in 1981 2,3 and the isolation of HIV-1 two years later 4, the disease has spread relentlessly, infecting close to 80 million people worldwide. The HIV epidemic, which was initially discovered and established in heterosexual populations of Central and East Africa 5,6, arose from zoonotic transmission of simian immunodeficiency virus (SIV) from non-human primates 7, suggesting a much older history of the pandemic 8. Using statistical approaches on HIV-1 sequence data from Central Africa, it was recently shown that the HIV-1 pandemic ignited in Kinshasa around the early 1920s and that its expansion in Central Africa was contingent upon an active transportation network connecting the country’s main population centers to other regions of sub-Saharan Africa 8.

Based on their genetic make-up, HIV-1 viruses are divided into four groups and represent three separate transmission events from chimpanzees (M, N, and O) and one from gorillas (P). Groups N (non-M non-O), O (outlier), and P are restricted to West Africa 9. Group M (major), which is the main cause of the global HIV pandemic, has diversified into nine subtypes (A-D, F-H, J and K), sub-subtypes (A1-A4 and F1 and F2), and numerous circulating (CRF) and unique recombinant strains (URF). Due to subsequent evolution and spread in the human population there are currently more than 60 CRFs (e.g. CRF01_AE and CRF02_AG) and numerous URF circulating 10. Subtype C predominates in the actual HIV-1 pandemic with a prevalence of almost 50% followed by subtype A (12%), subtype B (11%), CRF02_AG (8%), CRF01_AE (5%), subtype G (5%) and subtype D (2%). All other subtypes and CRFs represent about 5% of HIV-1 infections in the world 10.

The genetic diversity of HIV is primarily caused by the fast replication cycle of the virus coupled with the high error-prone function of its reverse transcriptase 11. These features allow HIV to evolve around one million times faster than mammalian DNA 12. Additional genetic diversity is introduced as a result of recombination that takes place during HIV replication when the host cell is infected with multiple HIV-1 subtypes, also known as co-infection or super-infection 13. Recombination allows for a more rapid increase in viral diversity than does the accumulation of mutations through replication errors. This genetic heterogeneity allows for rapid adaptation to host immune responses, target cell availability, and antiretroviral therapy, which can lead to increased viral pathogenicity, infectivity, and decreased antiretroviral susceptibility 13. Emerging evidence suggests that clinical progression to AIDS might be more rapid in individuals with dual infection 14.

To date more than 40 million people have died due to AIDS-related causes since the pandemic began and millions more are newly infected with the virus each year. In 2014, nearly 37 million people were infected with HIV and the number of people living with HIV continues to increase, in large part because more people globally have access to antiretroviral therapy (ART) 15. Particularly in the last decade there are signs that the pandemic may be changing course as new HIV infections and AIDS related deaths have significantly declined, contributing to an overall stabilization of the pandemic 16. For example, as of June 2015, 15.8 million people living with HIV were receiving ART representing over 41% of those in need. Significant progress has also been made in the prevention of mother to child transmission (MTCT) of HIV as 73% of pregnant women living with HIV had access to preventive treatment to protect their babies from infection. Moreover, global incidence has fallen from 3.1 million infections in 2000, to 2 million infections in 2014, representing a decrease of 35% in new infections. Notably, new HIV infections among children have declined by 58% since 2000 15 and AIDS related deaths have fallen by 42% since the peak in 2004. However, there is still an unacceptably high number of new HIV infections and AIDS-related deaths occurring each year. Alone in 2014, an estimated 2 million people became newly infected with HIV and 1.2 million died of AIDS-related illnesses 17.

**Figure 1:**
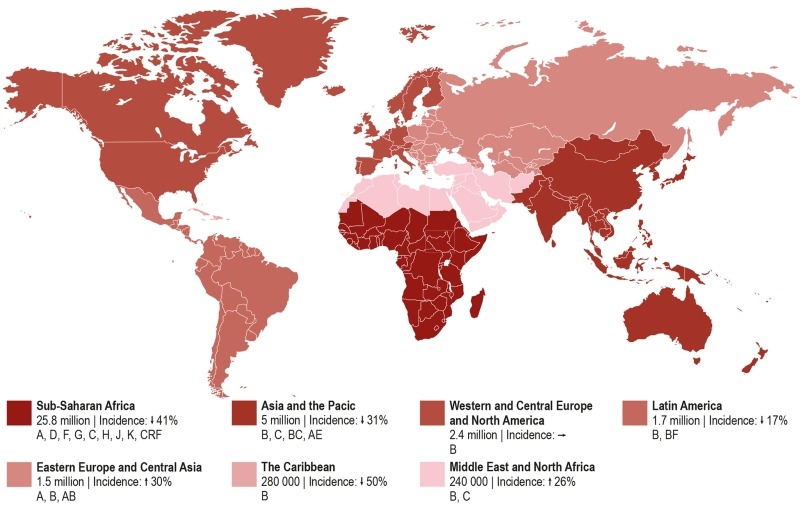
FIGURE 1: Worldwide distribution of estimated HIV-1 infections in 2014, trends in the incidence of new infections from 2000 to 2014, and HIV-1 subtypes. Numbers and percentages based on UNAIDS fact sheet 2015.

Today, there is no region of the world untouched by this pandemic (Figure 1). Spread of the disease has been particularly alarming in resource-limited countries, especially sub-Saharan Africa and Southeast Asia, but continues to threaten other populations in Eastern Europe, Latin America, and the Caribbean. Nearly 70% of the world’s HIV-infected population lives in sub-Saharan Africa and besides the Caribbean have the highest national rates of adult HIV prevalence (4.7% and 1.1%, respectively) 18. While the vast majority of new HIV infections in sub-Saharan Africa occur in adults over the age of 25 through heterosexual transmission, HIV disproportionately affects young women 19. More than 4 in 10 new infections among women are in young women aged 15-24 20. The HIV prevalence among females aged 15-19 is eight times higher than that among males at the same age 16. Sex workers, men who have sex with men (MSM), people who inject drugs (PWID), and children are also key affected populations in sub-Saharan Africa 21. The highest infection rates were reported for sex workers with an average HIV prevalence of 20%, compared to 3.9% globally and MSM with an HIV prevalence of 15% across Western and Central Africa and 14% across Eastern and Southern Africa 18.

Asia and the Pacific represent the second most affected regions by HIV with 14% of the world’s HIV-infected population. However, the region has made tremendous progress in tackling the HIV pandemic reducing the number of new infections by 31% since 2000. Also the adult HIV prevalence rate with 0.2% is relatively low compared to other regions like Western and Central Europe and North America (0.3%), Latin America (0.4%), and Eastern Europe and Central Asia (0.6%) 22. Risk groups include MSM, PWID, and transgender (TG) populations. The HIV prevalence for the latter population in numerous cities (e.g. Delhi, Phnom Penh, and Mumbai) is much higher than the HIV prevalence in MSM populations. However, MSM remain one of the key affected populations in Asia and the Pacific with rising HIV prevalence 22.

North America and Western and Central Europe constitute the third most affected region by the HIV pandemic with 2.4 million people living with HIV. The United States accounts for the majority of people living with HIV in this region (56%). Four countries of Western Europe including France (8%), Spain (6%), United Kingdom (5%), and Italy (5%) contribute an additional quarter of this number 18. The modes of transmission vary greatly between countries. For example, in 2014, MSM accounted for 44% of new HIV diagnoses in Western Europe and 28% in Central Europe. By comparison, PWID in Central Europe accounted for 5% of new HIV infections compared with 3% in Western Europe. Key affected populations in Western and Central Europe include MSM, migrants from sub-Saharan Africa, PWID and their sexual partners, transgender people, prisoners and sex workers are also at a heightened risk of HIV. In the USA, the majority of newly diagnosed HIV infections in 2013 among adult and adolescent males and females were attributed to MSM and PWID (68%). In contrast, heterosexual contact is thought to contribute 25% of new infections in the United States. African Americans are at a high risk of contracting HIV. In 2013 the infectivity rates were 60% for this racialized group.

The Middle East and North Africa has one of the world lowest HIV prevalence rates with 0.1%. However, new HIV infections have risen by 26% since 2000 and AIDS-related deaths increased by 66% since 2005, largely due to the fact that this region has the lowest ART coverage of any region in the world at 11% 18.

Recent trends in hospital deaths among HIV-infected patients showed that mortality during ART is often caused by diseases and conditions other than AIDS. According to a recent study, in-hospital deaths among HIV-infected patients declined significantly, and deaths that were not attributable to AIDS increased from 43.0 to 70.5% 23. Patient factors that were significantly associated with non-AIDS deaths versus AIDS-related deaths included older age (median age, 48 versus 40 years), more likely to be on ART (74.1 versus 55.8%), less likely to have a CD4 count of <200 cells/mm^³^ (47.2% versus 97.1%), and more likely to have an HIV viral load of <=400 copies/mL (38.1 versus 4.1%). The most common causes of non-AIDS deaths are non-HIV infection (20.3%), cardiovascular conditions (11.3%), liver disease (8.5%), and malignancies (7.8%) 23. Notably, the risk of myocardial infarctions in HIV infected people is 50% higher than in people without HIV 24. In addition, co-infection with hepatitis B (HBV) and C (HCV), which share similar routes of transmission with HIV, is more likely in HIV infected people 25. For example co-infection with HIV and HCV is very common (50%-90%) among HIV-infected injection drug users in the US 26.

HIV associated tuberculosis (TB) remains a global public health challenge among people living with HIV, accounting for around one in three AIDS-related deaths worldwide. A retrospective cohort study conducted in South Africa revealed that TB doubled within the first year after HIV infection 27, thereafter the incidence increased as immunity decreased, and reached a very high prevalence of 25.7 per 100 person-years in patients with CD4 T-cell counts lower than 50 cells per μL 28. However, TB-related deaths in people living with HIV have fallen by 32% since 2004 29.

## ETIOLOGY, TRANSMISSION AND PROTECTION

**Figure 2:**
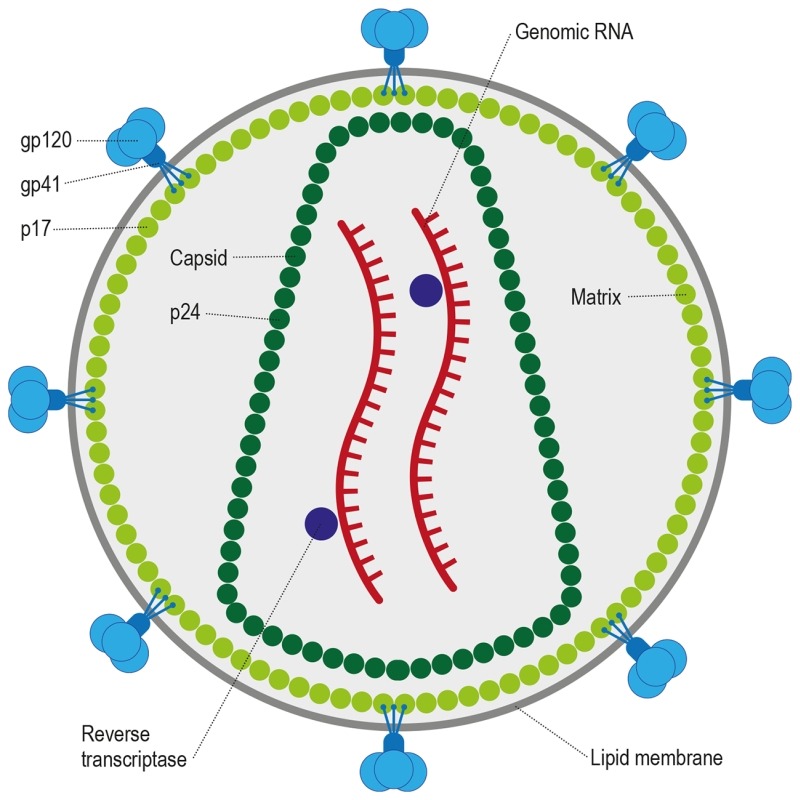
FIGURE 2: HIV-1 is a retrovirus that is approximately 90 - 120 nm in diameter and is enveloped by a host-derived plasma membrane. Trimeric envelope glycoproteins gp120/41 form the spikes on the virions surface and are embedded in the membrane. The cytoplasmic tail of gp41 interacts with the HIV-1 matrix protein p17. During maturation the capsid protein, p24, makes up the cone-shaped core, which contains two positive-strand RNA copies of the HIV-1 genome, the reverse transcriptase protein, as well as a number of other important host proteins.

**Figure 3:**
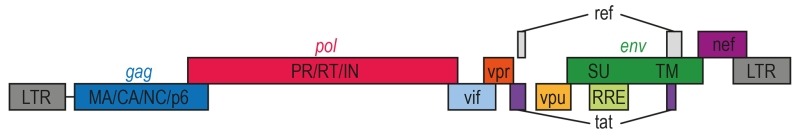
FIGURE 3: HIV-1 genome. A schematic representation of the HIV-1 gene products encoded by the HIV-1 genomic sequence.

HIV, the causative agent of AIDS, belongs to a class of viruses known as retroviruses and a subgroup of retroviruses known as lentiviruses or "slow" viruses 30. HIV is an enveloped, single-stranded positive-sense RNA virus (Figure 2) with a genome of 9749 nucleotides in length that encodes a total of nine viral proteins 31. The HIV genome contains three major genes including gag, pol, and env, encoding major structural proteins as well as essential enzymes (Figure 3). The gag gene encodes viral core proteins, the pol gene encodes a set of enzymes required for viral replication, and the env gene encodes the viral surface glycoprotein gp160 32. In addition to these three major proteins, HIV also encodes proteins with certain regulatory and auxiliary functions containing Tat and Rev, which activate viral transcription and control the splicing and nuclear exports of viral transcripts, respectively 33. Four other genes encode accessory proteins Vif, Vpr, Vpu and Nef, which are not essential for replication in certain tissues. The viral genome is flanked by LTRs (long terminal repeats) that are required for viral transcription, reverse transcription and integration (Figure 3) 34. The genome dimerization and packaging signal ‘Ψ’ is located between the 5'-LTR and the gag gene 35.

**Figure 4:**
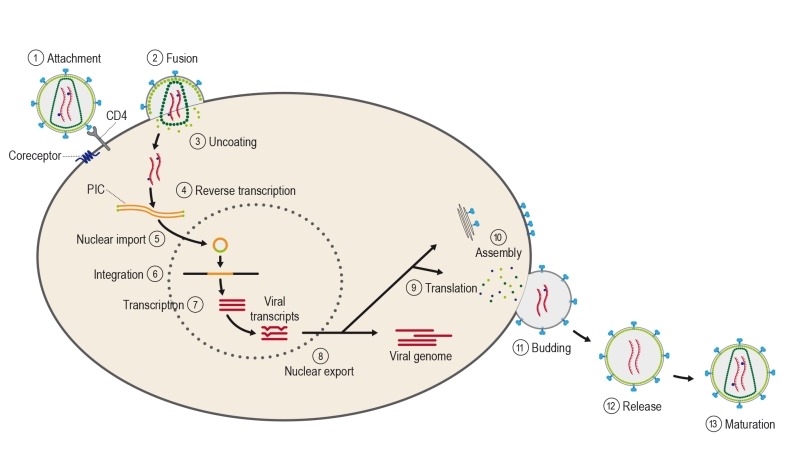
FIGURE 4: Different steps of the viral life cycle. The infection cycle begins with the attachment of the envelope (Env) glycoprotein spikes with the CD4 receptor and the membrane-spanning coreceptor (step 1), leading to fusion of the viral and cellular membranes and entry of the viral particle into the cell (step 2). Partial uncoating (step 3) facilitates reverse transcription (step 4), which in turn yields the pre-integration complex (PIC). Following import into the cell nucleus (step 5), PIC-associated integrase orchestrates the formation of the integrated provirus (step 6). Proviral transcription (step 7) yields viral messenger RNAs (mRNAs) of different sizes. Following export (step 8), mRNAs serve as templates for protein production (step 9), and genome-length RNA is incorporated into viral particles with protein components (step 10). Viral-particle budding (step 11) and release (step 12) is accompanied or soon followed by protease-mediated maturation (step 13) to create an infectious viral particle.

The course of infection with these viruses is typically characterized by a long period between initial infection and the onset of serious symptoms. Like all viruses, HIV can reproduce only inside cells by hijacking the cell’s machinery (Figure 4). Once inside the cell, HIV and other retroviruses use the enzyme reverse transcriptase (RT) to convert their viral RNA into DNA, which can be incorporated into the host cell genome 36. Once integrated, the proviral DNA is replicated along with cellular DNA during cycles of cell division, as with any cellular gene. The provirus serves as the template for transcription of viral RNAs. Some viral RNAs are translated to yield the viral proteins, whereas a portion of the full-length viral RNA is recruited to serve as genomic RNA in progeny virions 37.

Transmission of HIV requires contact with a body fluid that contains either infectious virus (virions) or HIV-infected cells or a combination of both 38. HIV can appear in nearly any body fluid 39, but transmission occurs predominantly through blood, semen, vaginal and rectal fluids, and breast milk 40. Although tears, urine, and saliva may contain low concentrations of HIV, transmission through these fluids is extremely rare, if it occurs at all. No case of HIV transmission has been traced to the coughing or sneezing of an infected person or to a mosquito bite. The three main routes of HIV transmission are parenteral exposure (e.g. blood transfusion, needle sharing), unprotected sexual contact, and vertical (mother to child) trans-mission 41. Sexual exposure is the most common route of infection and drives the HIV pandemic in most countries, followed by needle sharing injective drug use, and MTCT 42. Based on a recent study 43 analyzing the per-act HIV transmission risk estimates (Table 1) the authors found that blood transfusion ranked on top, followed by vertical transmission, receptive anal intercourse, needle-sharing injection drug use, percutaneous needle stick injuries, insertive anal intercourse, receptive penile-vaginal intercourse, and insertive penile-vaginal intercourse. Although biologically plausible, the transmission risk for receptive and insertive oral sex is relatively low as the oropharynx is considerably less susceptible to HIV infection than the cervico-vaginal environment or penis. This might be due to the thicker epithelial layer of the oropharynx, the low number of CD4^+^ lymphocytes, and the presence of HIV-specific antibodies and various endogenous factors that inhibit HIV transmission 40,43.

**Table 1:** Estimated per-act HIV transmission risk per 10,000 exposures. Adapted from 43. ^a^ Risk is considered to be low relative to the other sexual exposures, but it is not zero.

**Route of transmission**	**Infection risk**	**95% Confidence interval**
Blood Transfusion	9250	8900-9610
Mother to Child	2255	1700-2890
Receptive Anal Intercourse	138	102-186
Drug Use Needle Sharing	63	41-92
Percutaneous Needle Stick	23	0-46
Insertive Anal Intercourse	11	4-28
Receptive Penile-Vaginal Intercourse	8	6-11
Insertive Penile-Vaginal Intercourse	4	1-14
Insertive Oral Sex	Low^a^	0-4

Anal intercourse carries a higher risk of HIV transmission for both receptive and insertive partners when compared with vaginal intercourse 44. The risk of HIV transmission to the receptive partner resulting from receptive anal intercourse is almost 18 times higher than the risk from receptive vaginal intercourse 45. The higher risk of infection is thought to result from the differences between the tissues involved. First, rectal mucosa is characterized by a higher density of lymphoid follicles, which are overlaid with microfold cells (M cells) that are specialized in antigen uptake. Second, M cells form intraepithelial pockets containing CD4^+^ memory T cells, macrophages, and dendritic cells (DCs) in close proximity, which could greatly facilitate HIV replication. Third, the single layer of epithelial cells in the rectum could be more susceptible to abrasions than the vaginal mucosa 46,47.

One of the most important factors that increase the risk of sexual transmission of HIV-1 is the viral load (i.e. number of viral RNA copies per mL of plasma) 48,49. During primary infection, the number of HIV-1 particles in plasma increases rapidly, reaches a peak (median 5.8 log10 HIV-1 copies/mL), and then declines until it reaches a set point level 50. It has been reported that the per act risk of heterosexual transmission of HIV in serodiscordant couples is 2.9 fold (95% CI, 2.2-3.8) increased for each 1.0 log10 increase of the viral load. In contrast, a reduction in plasma viral load of 0.7 log10 is estimated to reduce HIV-1 transmission by 50% 51. Moreover, higher genital HIV-1 RNA concentrations are also associated with greater risk of heterosexual HIV-1 transmission, and this effect is independent of plasma HIV-1 concentrations, which would make HIV-1 RNA in genital secretions a useful marker of HIV-1 sexual transmission risk 49. The stage of infection is also an important variable for infectivity, largely because of the accelerated rate of viral shedding during the acute stage 52. Viral loads in all fluids and tissues, including blood and genital secretions, peak around 4 weeks after viral exposure. The risk of sexual transmission of HIV during this stage is 30-300 times the risk during the post-acute phase of infection, when antibodies and cytotoxic T cell lymphocytes directed against HIV appear 52. Based on a study conducted in Uganda with HIV-discordant couples 53 it was found that the rate of heterosexual HIV transmission per coital act was highest during early-stage infection, a time when only few seroconverters know their HIV status or receive ART. It is believed that patients with early-stage HIV infection make a highly disproportionate contribution to the incidence of HIV infection 52.

Sexually transmitted diseases (STDs) (e.g. genital ulcer disease of any cause, herpes simplex type-2 (HSV-2) infection, and bacterial vaginosis) can also greatly increase both infectivity and susceptibility of HIV 54. On the susceptibility side, STDs can reduce the efficacy of physical and mechanical barriers of the virus (e.g., by causing lesions or aberrations in the mucosa) 55, increase the number of HIV receptor cells or the number of receptors per cell (e.g. by causing persistent inflammation) 56, and produce a vaginal environment that is more conducive to transmission (e.g. via presence of bacterial vaginosis and increased levels of anaerobic bacteria or various amines) 57,58. On the infectivity side, STDs might evoke more infectious HIV variants and thus increased HIV concentrations in genital lesions, semen, or both 59. Moreover, co-transmission of HIV and another STD appears to be a common occurrence 60. Specific host factors may also augment the risk of HIV transmission 61. For example, the risk of HIV transmission is elevated 2-3 times in women with cervical ectopy, a condition that renders cervical tissues more vulnerable 62, while in men, the presence of foreskin has been associated with increased risk of HIV acquisition 63. In addition any factor that increases the opportunity for HIV to reach receptive immune cells may amplify the risk of HIV transmission. For example, hormonal contraceptives have been implicated in several studies, because of their association with vaginal thinning or cervical ectopy 64,65,66. Frequent use of spermicides containing N-9 has been associated with the disruption and irritation of the genital epithelium that may increase the risk of HIV infection 67,68. The presence of blood during sexual intercourse, including blood associated with menstruation, has been linked with an increased risk of HIV transmission, particularly from females to males 52.

Prevention of sexual HIV transmission has been a priority since the beginning of the epidemic. To control the epidemic many interventions are necessary as no isolated prevention intervention is effective enough on its own. Prevention interventions that can significantly decrease risk of viral infection include: 1) use of antiretroviral drugs for treatment, 2) pre-exposure prophylaxis (PrEP) and post-exposure prophylaxis (PEP), 3) male condom use, and 4) medical male circumcision 69,70,71. The most potent intervention to reduce sexual transmission of HIV is ART. Findings of the landmark HIV Prevention Trials Network (HPTN) 052 trial revealed that immediate ART treatment in serodiscordant couples reduced HIV transmission in the uninfected partner by 96% due to universal viral suppression 72. However, results of HPTN 052 also clearly displayed the demand for integral prevention interventions, as 25% of HIV transmissions were not from the HIV infected partner 72. A population-based prospective cohort study in rural KwaZulu-Natal, South Africa, with a total of 16,667 HIV negative participants demonstrated that the risk of HIV infection was associated with ART coverage in the local community 73. For example, an HIV-uninfected individual living in a community with high ART coverage (30 to 40% of all HIV-infected individuals on ART) was 38% less likely to acquire HIV than someone living in a community where ART coverage was low (<10% of all HIV-infected individuals on ART) 73. The population-level effects resulting from early ART initiation independent of CD4 counts, the so-called test and treat strategy, are currently being studied in several randomized trials 74. One of the studies reported a 90% reduction of HIV transmission due to early ART initiation among seronegative sex partners in stable or casual relationships with seropositive individuals. 75. The feasibility of achieving benefits of ART will need effective interventions to greatly increase the awareness of HIV status. HIV testing and counseling is the first crucial step for linkage to HIV treatment and prevention. However, conventional facility-based HIV-testing and counseling (HTC), although important, has not achieved high testing coverage in sub-Saharan Africa due to limitations in the health care system 76,77. A meta-analysis 78 found that community HTC had high coverage and uptake and identified HIV-positive people at higher CD4 counts than facility testing. Mobile HIV testing reached the highest proportion of men of all modalities examined (50%, 95% confidence interval (CI) = 47-54%), while self-testing at home reached the highest proportion of young adults (66%, 95% CI = 65-67%). Key populations (commercial sex workers and MSM) yielded high HIV positivity (38%, 95% CI = 19-62%) combined with the highest proportion of first-time testers (78%, 95% CI = 63-88%), indicating service gaps 78. Community testing with facilitated linkage (for example, counselor follow-up to support inclusion into the program) achieved high access to care (95%, 95% CI = 87-98%) and antiretroviral initiation (75%, 95% CI = 68-82%). Expanding home and mobile testing, self-testing and outreach to key populations with facilitated linkage can increase the proportion of men, young adults and high-risk individuals linked to HIV treatment and prevention, and decrease HIV burden 78. However, a cautionary point about the introduction of the test and treat strategy is that it could increase antiretroviral resistance 79. Moreover, optimism about the treatment or misperceptions about the effects of antiretroviral drugs may also cause some people to increase their risk behavior 80. Meta-analyses of studies conducted in several different risk groups (e.g. MSM, PWID, sex workers) revealed that behavioral interventions reduced self-reported risk behavior. For example, findings of a study of African heterosexual serodiscordant couples showed that self- reported condom use shortened the per-coital act risk of HIV transmission by 78%. 51 A combination of antiretroviral treatment and condom use could theoretically reduce the per-act HIV transmission risk of anal and vaginal intercourse by up to 99.2% 43. Disclosure of HIV status to sexual partner is an important prevention goal that increases the awareness of HIV risk to untested partners thus leading to greater acceptance of HIV counseling and testing as well as positive changes in risk behaviors 81. The effect of PrEP on HIV acquisition has been studied in several clinical trials 82,83,84,85. A once-daily oral dose of tenofovir (TFV) or TFV plus emtricitabine effectively reduced HIV acquisition by 44% to 75%. However, efficacy was strongly associated with adherence to the intervention program 86. A very recent "on-demand PrEP" study conducted in men who have unprotected anal sex with men revealed that a combination of TFV and emtricitabine, taken before and after sexual activity, provided notable protection against HIV acquisition with a relative reduction of 86% in the risk of HIV-1 infection 87.

Medical male circumcision (MC) on the other hand is not only cost saving but also very effective in reducing HIV acquisition in men. A randomized trial with circumcised men in Uganda revealed that HIV incidence over 24 month was 0.66 cases per 100 person-years in the intervention group (MC) versus 1.33 cases per 100 person-years in the control group who were not circumcised 88. A similar result was found in Kenya, where the risk of HIV acquisition of circumcised men was reduced by 53% 89. Furthermore, the rate of male-to-female HIV transmission after MC is reduced by 46% 90. MC also decreases HSV-2 and human papillomavirus (HPV) infection among heterosexual men and provides benefits to female partners, including reduced prevalence of genital ulcer disease, bacterial vaginosis, and HPV 91. Despite the potential public health benefits of medical MC, there are several scale-up challenges. Many strategies are needed to increase demand for medical MC, including promotion of benefits of circumcision to men and their female partners, and supply-side interventions to provide medical MC through mobile clinics and devices that reduce procedure time 91.

Topical PrEP application of vaginal and rectal microbicides is an attractive intervention because, unlike condoms, they are under the control of the receptive partner. Vaginal application of 1% TFV gel demonstrated complete protection from SHIV infection in macaques, when applied 30 min before viral challenge 92. However, to date there is no real evidence of protection against HIV with the exception of the findings of the CAPRISA 004 trial in South Africa where pericoital use of 1% TFV gel reduced HIV acquisition by 39% 93. Unfortunately, the result of the CAPRISA 004 study could not be reproduced in a more recent confirmatory trial called FACTS 001 94,95. Among the 2029 women in the study allocated to either TFV or placebo gel, there were 61 infections in women using TFV gel and 62 in women using placebo 95. The use of 1% TFV as a rectal microbicide -applied daily or before and/or after sex to prevent HIV- in MSM and transgender women was recently assessed in a phase 2 extended safety and acceptability study (MTN-017). Based on the results presented at CROI 2016 there was no difference in adherence between sex-based rectal gel use and a daily oral PrEP option 96. It remains to be seen in upcoming phase III clinical trials whether topical application of rectal microbicides result in a similar efficacy as orally administered PrEP or not. Newer strategies in microbicide development are focused on the sustained delivery of antiretroviral drugs. The dapivirine vaginal ring for HIV-1 prevention in women, which delivers low doses of the NNRTI dapivirine over a month of use, is one example 97. The efficacy of the dapivirine vaginal ring has been recently tested in a phase III clinical trial conducted in Malawi, South Africa, Uganda, and Zimbabwe. A total of 168 HIV-1 infections occurred among the 2629 women who were enrolled in the trial. The dapivirine group had a 27% lower incidence rate (3.3 cases per 100 person-years; 71 infections) than the placebo group (4.5 cases per 100 person-years; 97 infections) 98.

Other interventions to reduce HIV infectivity have focused on treatment of co-infections, notably HSV-2 infection, which causes genital herpes 99. Although aciclovir and valaciclovir treatment effectively reduce plasma and genital HIV concentrations no association with decreased HIV transmission has been found, except slightly delayed HIV disease progression 100. Additional research is needed to determine the aspect of alternative interventions to treat co-infections.

Substantial progress has been made in the risk reduction of perinatal HIV-1 transmission. MTCT of HIV is relatively rare during early pregnancy and relatively frequent in late pregnancy and during delivery. Knowledge about the timing of HIV-1 transmission to infants has allowed the development of appropriate interventions 99. Without any intervention, the estimated risk of perinatal transmission ranges from 15% to 40% 101, depending on maternal risk factors (e.g. plasma and breast milk viral load, maternal immunologic status and clinical stage) and whether breastfeeding is practiced 102. In sub-Saharan Africa, where prolonged breastfeeding is customary, breast milk transmission represents an important mechanism of MTCT of HIV-1 103. In a data meta-analysis of more than 3,000 breastfeeding infants of HIV-1-infected women from sub-Saharan Africa, rates of HIV infections through breast milk were estimated with relatively high precision. The study found that the cumulative probability of late postnatal transmission at 18 months was 9.3% and the overall risk of late postnatal transmission was 8.9 transmissions per 100 child-years of breastfeeding 104. Whether breast-feeding is "exclusive" or "mixed" has also been shown to be of particular importance in the risk of MTCT. Exclusive breast-feeding has been found to have a significantly lower transmission risk than mixed feeding 101. This is thought to be due to a disruption in the integrity of the intestinal mucosa, which is normally protected by breast milk, allowing HIV to more readily penetrate these microabrasions 105.

Analyses of viral load levels from several studies demonstrated a direct positive correlation between maternal RNA viral load in plasma and risk of transmission to the infant 106. High levels of virus in plasma, and presumably also in breast milk, are observed in primary HIV infection, when the rate of postnatal transmission has been estimated to be nearly 30% 107. This correlation is also observed among women receiving ART. For example, in a study conducted in the United Kingdom and Ireland with over 10,000 HIV-positive pregnant women, MTCT rates were lower among women who had a viral load <50 copies/mL near delivery, compared with women who had higher viral loads (0.09 percent transmission versus 1.0 and 2.6 percent with viral load ranges 50-399 copies/mL and 400-999 copies/mL, respectively) 108.

Additional maternal and infant factors that have been associated with higher risk of transmission are 1) low CD4 cell counts, 2) anemia, 3) more advanced WHO clinical disease stage, 4) maternal mastitis, and 5) acute maternal seroconversion during pregnancy or breastfeeding 109,110. As an example, in a recent meta-analysis, which included data from 19 cohorts and 22,803 total person-years, the pooled HIV incidence rate during pregnancy/postpartum was 3.8 events per 100 person years, and the pooled risk of MTCT among such women was 23 percent 111. Recommended MTCT interventions, predominantly ART, have resulted in a ten-fold reduction in this risk, and complete elimination of MTCT is now feasible 99. Antiretroviral combination therapy is more effective at prevention of MTCT than zidovudine plus one dose of nevirapine, and has the additional advantages of reducing sexual HIV transmission and reducing HIV-associated morbidity and mortality 99. ART treatment should ideally be started after the first trimester, provided that women are well enough to delay their treatment 112. However, ART alone will not reach the goal of elimination of prevention of MTCT. Access to prenatal care, HIV testing, and MTCT interventions will need to be substantially increased in regions with high HIV prevalence 112.

Despite a number of successful prevention interventions that have been reported, including PrEP prophylaxis and treatment as prevention, ultimate control of the HIV epidemic will most likely come only with the development of a safe and effective preventive vaccine. However, this goal has proved to be elusive 113. Of the reported HIV vaccine efficacy trials 114,115,116,117,118 only the RV144 HIV vaccine trial consisting of a recombinant Canarypox virus based vector (ALVAC-HIV) and a recombinant envelope glycoprotein gp120-subunit vaccine (AIDSVAX B/E) has been successful in reducing HIV incidence by 31% 119,120,121. In contrast, the HVTN 505 vaccine study consisting of a DNA prime-recombinant adenovirus type 5 boost (DNA/rAd5) regimen conducted in the US revealed a vaccine efficacy of -25% after a first evaluation with 27 infections in the vaccine group and 21 infections in the placebo group 113. The data and safety monitoring board stopped further vaccinations due to the fact that the DNA/rAd5 vaccine regimen did not reduce either the rate of HIV-1 acquisition or the viral-load set point in the population studied 113. Nevertheless, the partial success of the RV144 study has refueled the vaccine field and has led to the development of a vaccine protocol (HIV Vaccine Trials Network (HVTN) 100) to assess the efficacy of this strategy against clade C HIV in South Africa sponsored through the National Institute of Allergy and Infectious Diseases.

## PATHOLOGY/SYMPTOMATOLOGY

HIV-1 infection is usually initiated with a single virion infecting a single target cell at the site of entry. Mucosal surfaces represent the number one entry site of HIV as the vast majority of HIV infections result from mucosal transmissions 122. Mucosal transmission is dependent upon transfer of infectious virus and/or cells across the mucosal epithelium providing access to sub-epithelial DCs, macrophages, monocytes, and/or CD4 T-cells 123,124. CD4-independent HIV infection of cells has been reported in several occasions involving astrocytes and renal epithelial cells where HIV gene expression plays an important role in the pathogenesis of HIV-associated neurocognitive disorder related to astrocytes and nephropathy related to epithelial cells 125,126.

Numerous mechanisms for mucosal HIV-1 transmission have been proposed including: (i) direct HIV-1 infection of epithelial cells, (ii) transcytosis of HIV-1 across epithelial barriers and/or specialized M cells, (iii) epithelial transmigration of HIV-1-infected donor cells, (iv) uptake of HIV-1 by intra-epithelial Langerhans and dendritic cells, (v) or entry via epithelial micro-abrasions or ulceration 127,128. Non-human primate studies have revealed that mucosal infection can occur within a very short time period. Following 30-60 min exposure to infectious virus, local infection is established within 16-72 hours, and expansion to draining lymph nodes is accomplished within 24-72 hours 129,130.

Establishment of HIV infection across mucosal membranes most often results from the transmission and subsequent propagation of a single virus strain, termed transmitted/founder (T/F) virus 131. This was demonstrated by the isolation of a single viral genome and its unique viral envelope (Env) in 102 acute HIV-1 infected subjects, where 78 subjects had evidence of productive clinical infection by a single virus, whereas the remaining 24 subjects were infected by a minimum of two to five viruses 132.

Several distinctive phenotypic characteristics of T/F virions are clearly associated with transmission: Chemokine receptor 5 (CCR5) tropism, CD4^+^ T cell tropism, enhanced cell-free infectivity, higher Env content, improved DC interaction, and relative IFN-α resistance 133,134,135,136. Other phenotypic and genotypic attributes that have been linked to transmission are: Enhanced CD4 receptor and/or coreceptor engagement, increased sensitivity to neutralizing antibodies, Env interaction with integrin pair-α4β7, fewer putative N-linked glycosylation sites, and shorter variable loops 137,138,139,140,141,142,143. However, to date, no consistent phenotypic correlate of these genetic signatures has been identified 133,144.

**Figure 5:**
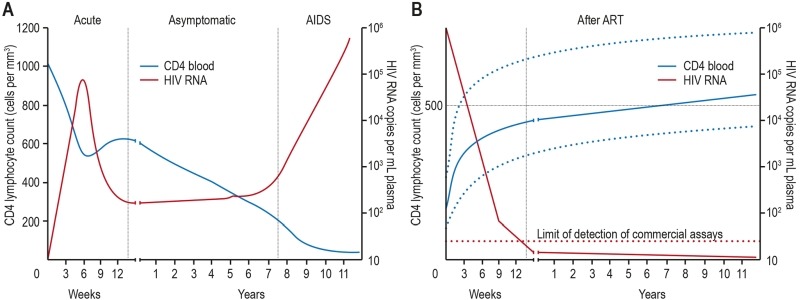
FIGURE 5: The course of untreated HIV infection and changes after antiretroviral therapy. In untreated HIV infection the blood CD4 T cell count progressively declines over the course of infection **(A)**. After initiation of antiretroviral therapy the HIV RNA copy numbers significantly decrease below detection limit followed by recovery of CD4 T cells, which can vary notably between individuals **(B)**. Adapted from 99.

Although a typical time course of infection can vary considerably from individual to individual, as can the levels of viremia, the general outline is essentially the same in virtually every infected person who does not receive effective ART therapy (Figure 5A) 145. In the eclipse phase, 1-2 weeks after infection, the T/F virus is freely replicating and spreading from the initial site of infection to the many tissues and organs that provide the sites for replication. At that stage viremia is still undetectable, and neither immune response nor symptoms of infection are yet visible. The next phase, also called acute or primary infection phase, 2-4 weeks after infection, is characterized by relatively high levels of viremia (up to 107 or more copies of viral RNA/mL of blood) and large fractions of infected CD4^+^ T cells in blood and lymph nodes. The high levels of viremia most likely result from the absence of the early immune response and the generation, as part of the host response, of large numbers of activated CD4^+^ T cells, thus providing extra targets for viral replication 145. The rapid increase in HIV replication follows a striking induction of inflammatory cytokines and chemokines, which is in complete contrast to the minimum initial response to other chronic viral infections such as hepatitis B or hepatitis C 99,146.

The acute phase is often, but not always accompanied by "flu-like" symptoms including fever, sore throat, lymphadenopathy, and rash 147. Around the time of peak viremia, the immune response begins to appear, both humoral (antibodies response against all viral antigens) and cellular (CD8^+^ T-cell response against HIV-1 antigens expressed on infected cells) 148. The CD8 mediated killing of productively HIV-infected cells and the potent adaptive immune response to HIV, both select for the emergence of mutations in key viral epitopes which often leads to immune escape and so called highly diverse HIV quasispecies 99,149,150. In certain cases, overrepresentation of specific HLA class I alleles (e.g. HLA-B27) is associated with an effective immune response, characterized by HIV-specific T cells with high avidity, polyfunctionality, and capacity to proliferate against both the immunodominant epitopes (e.g. for HLA-B27 the B27-KK10 epitope (KRWIILGLNK at positions 263-272) in p24 Gag) and escape variants thereof 151,152,153. HLA alleles HLA-B57 and HLA-B5801 also exert strong selection pressure on the virus and are thus associated with long-term HIV control 154. Interestingly, although HLA-B57 is correlated with slow progression to disease following HIV-1 infection, B-57 heterozygotes display a wide spectrum of outcomes, including rapid progression, viremic slow progression, and elite control 155.

However, only about 3% of the general population possess these specific HLA class I alleles, while in nearly all individuals a progressive exhaustion of HIV-specific CD8^+^ T cells occurs. This is characterized by an upregulation of the gene programmed death 1 (PD-1) expression on both total and HIV-specific CD8^+^ T cells and a loss of effector function. In addition, it was found that PD-1 expression levels correlate significantly with viral load and with the reduced capacity for cytokine production and proliferation of HIV-specific CD8^+^ T cells 156. In contrast, CD8^+^ T-cells that see HLA B27/B57 cells in long-term nonprogressors do not get exhausted which helps these individuals to control the disease 157. Similar studies in SIV infected rhesus macaques revealed that expression of the MHC class I alleles Mamu-B*08 and Mamu-B*17 correlates with viral control 158,159.

Autologous neutralizing antibodies arise roughly 3 months after infection 160. The typical course of infection is characterized by the development of autologous antibody responses capable of neutralizing virus, selection of escape variants, development (after a delay) of new responses capable of neutralizing the new escape variants, and selection of new escape variants, with the host always playing catch-up 148,161. Broadly neutralizing antibodies, which can neutralize many HIV-1 subtypes, are produced by about 20% of patients 162. In addition, 2-4% of these patients have even greater serum neutralizing activity that inactivates most HIV-1 strains from different clades 163. These antibodies are characterized by a high frequency of somatic mutations that often take years to develop 164. However, as described above broadly neutralizing antibodies do not usually provide benefit to the patient because of the development of viral escape mutants. Nevertheless, antibodies also mediate important effector functions next to neutralization like antibody dependent cellular cytotoxicity (ADCC) 165,166, antibody-dependent cell-mediated virus inhibition (ADCVI) 167,168 or antibody-mediated phagocytosis 169,170. Antibody effector functions mediated through Fc binding are thought to be one possible mechanism mediating protection from HIV-1 infection in humans in the recent Thai RV144 vaccine efficacy trial 121. The production of broadly neutralizing antibodies by use of new immunogen design strategies is a major focus of vaccine research 171,172,173. At the end of the acute phase the viral load decreases sharply, 100-fold or more, a result, which is established largely by innate and adaptive immune responses as well as exhaustion of activated target cells (i.e. transient decline in the CD4^+^ T cell number/mL blood) 145.

The chronic infection, or "clinical latency" (1-20 years after infection) is characterized by a constant or slowly increasing level of viremia (1-1x10^5^ copies/mL), also called the "set point", and steady, near normal (around 1,000 cells/mL) or gradually falling levels of CD4^+^ T cells. Usually no symptoms are shown during that phase making infected people unaware of their status 145. Despite the term "latency", the viral infection is far from latent, with large numbers of CD4^+^ T cells becoming infected and dying every day. Finally, the number of CD4^+^ T cells declines to the point (around 200 cells/mL) at which immune control of adventitious infectious agents can no longer be maintained, and opportunistic infections begin to appear 145. Infections attributable to organisms such as *Pneumocystis jirovecii*, mycobacteria, cytomegalovirus, *Toxoplasma gondii*, and Cryptococcus as well as the occurrence of malignancies related to viral pathogens such as non-Hodgkins lymphoma and Kaposi’s sarcoma are common 174,175,176. Nonetheless, the profound immune deficiency also affects humoral defenses, placing infected persons at increased risk for infection with pathogens like *Streptococcus pneumoniae* 176,177. Control of the HIV-1 infection itself is also lost, and the level of viremia rises during the AIDS phase, culminating in death of the infected patient. Indeed, untreated HIV-1 infection is one of the most uniformly lethal infectious diseases known, with a mortality rate well over 95% 145.

Despite profound immune deficiency, there is evidence of profound immune activation in HIV infection as T lymphocytes, B-lymphocytes, and antigen-presenting cells of the innate immune system have phenotypic and functional evidence of activation 178,179. In acute infection, a massive increase of cytokines, called cytokine storm, is a characteristic of HIV-1 infection 146 and the levels of some of these cytokines are predictive of the rate of CD4^+^ T-cell loss and of T-cell activation levels 180,181. For example, CD8^+^ T cells often express high levels of activation markers such as CD38 and HLA-DR, which correlate with the viral load in non-controllers. Moreover, markers linked to immune senescence such as CD57 182 and immune exhaustion such as PD-1 156,183 are also elevated, and cells expressing each of these markers have demonstrable impairments in response to T cell receptor stimulation. Plasma levels of the IFN-inducible protein-10 (IP-10) during acute infection are predictive of rapid disease progression 181, while the frequency of CD8^+^ CD38^+^ cells is a valuable predictor of disease outcome in HIV infection and correlates well with the viral load 184. Likewise, high levels of inflammatory and coagulation markers predict morbidity and mortality in treated HIV infection. One of the hallmarks of immune activation in HIV infection is the progressive depletion of circulating T cells by activation-induced cellular turnover 185,186,187. This increased cellular turnover is seen in both CD4 and CD8 T-cell populations 187 and is especially remarkable among central memory cells in both humans and in SIV-infected macaques 188,189. Activated cycling CD4^+^ T cells are more susceptible to productive HIV infection 190,191 and also tend to die *ex vivo*, likely as a result of apoptosis 192.

Several potential drivers have been postulated to account for systemic immune activation in progressive HIV and SIV infection including the virus itself, which can drive activation of innate immune receptors such as TLR 7 and 8 through poly(U)-rich sequences in its genome 193,194 as well as possibly through activation of other innate immune receptors by capsid proteins 195 or viral DNAs 196. A partial decrease in the abnormal cytokine profile is observed after the administration of highly active ART, which might contribute to lower levels of HIV replication and restoration of CD4 T cell counts 197. Some level of T-cell activation in HIV infection might be also induced by the interaction of either HIV specific peptides or peptides from opportunistic microbes (such as cytomegalovirus and other herpes viruses), that have been permitted to replicate more effectively in the setting of HIV-related immune deficiency, with toll-like receptors (TCRs) 198. It is also possible that some level of immune activation in HIV and pathogenic SIV infection is related to homeostatic mechanisms, that is, a need to replenish lymphocyte populations at effector sites of potential microbial invasion 199. Additionally, there is increasing evidence that in HIV and in pathogenic SIV infection, early damage to mucosal CD4 T-cell defenses permits increased translocation of microbial products from the gut to the systemic circulation 200 and these microbial products can drive T-cell and innate immune cell activation 200,201.

In contrast, natural SIV hosts like sooty mangabeys, African green monkeys and mandrills share many features of HIV infection of humans; however, they usually do not develop immunodeficiency 202. Interestingly, both innate and adaptive immune activation are observed during the acute phase of SIV infection of natural hosts 203,204 including strong upregulation of type I interferon-stimulated genes in both peripheral blood and lymph nodes 205 as well as type 1 interferon (IFN-1) production by plasmacytoid DCs in lymph nodes 206. However, marked differences in the levels of immune activation between natural and non-natural HIV and SIV hosts are observed after the transition from the acute to the chronic phase of infection. Natural, nonprogressive SIV infections represent an evolutionary adaptation that allows a peaceful coexistence of primate lentiviruses and the host immune system. However, this adaptation does not result in reduced viral replication but, rather, involves phenotypic changes to CD4^+^ T cell subsets, limited immune activation and preserved mucosal immunity, all of which contribute to the avoidance of disease progression and, possibly, to the reduction of vertical SIV transmission 202.

High levels of circulating plasma IFN-1 in early HIV infection but not chronic HIV infection can suppress HIV replication and promote maturation and differentiation of antigen presenting cells such as DCs, monocytes, and macrophages 207. In several studies of a cohort of HIV-exposed seronegative (HESN) commercial sex workers from Nairobi, Kenya, it could be demonstrated that qualitative differences in CD4^+^ T cell responses and HIV-specific CD4^+^ and CD8^+^ T cells, as well as genetic factors such as enrichment of certain HLA alleles and haplotypes near the IFN regulatory factor 1 gene have been associated with protection 208,209,210,211,212,213. Furthermore it was shown that these individuals produce lower levels of proinflammatory cytokines at baseline than HIV-negative control subjects. Moreover, CD4^+^ T cells of these HESN commercial sex workers have a characteristically lower level of expression of genes and systems crucial for HIV replication, such as the T cell receptor pathway and previously identified HIV dependency factors 214. This apparent lowered activation results in "immune quiescence", which may contribute to host resistance to HIV 214. Finally, data from two other groups have also shown an association between reduced T cell activation and decreased HIV susceptibility. Studies of highly exposed MSM showed that HESN individuals had lower levels of CD45RO^+^ memory T cells and a lower percentage of activated CD4^+^ T cells than HIV-susceptible control subjects 215. Similarly, a study of HESN commercial sex workers from Côte d'Ivoire demonstrated lower expression of the activation marker CD69 in memory subsets of CD4^+^ and CD8^+^ T cells after alloimmune stimulation 216.

## TREATMENT AND CURABILITY

HIV infection has developed from a fatal into a manageable chronic disease with life expectancy, in some instances, estimated to be near that of the general population 217. Treatment with ART is life-long as it only suppresses the replication of HIV but does not eradicate or cure the infection 218. Stopping ART results in HIV viral load rebound, progressive CD4^+^ T-cell count decline, and clinical disease progression 219. One of the primary aims of ART treatment is to maintain health by preventing clinical disease progression at low cost of drug toxicity 220. This is achieved by inhibiting viral replication by antivirals, resulting in long-term suppression of plasma viral load 221. Treatment success is defined as maintaining plasma viral load at an undetectable level (<50 copies/mL) and by reconstitution of the immune system (Figure 5B) 222. A secondary aim is prevention of HIV transmission by ‘treatment as prevention’ as described earlier 223. Unfortunately, there is only limited evidence available from randomized trials to guide the decision to start therapy in naıve individuals. However, a recent study investigated the rate of new infections during administration of PrEP in a clinical practice setting. Despite high rates of sexually transmitted infections among PrEP users and reported decreases in condom use in a subset, no new HIV infections in this population were reported 224.

Based on evidence from observational studies, current clinical guidelines all recommend that ART should be initiated if the CD4^+^ count is lower than 350 cells/mm^3^ or if an opportunistic infection has been diagnosed 225. In contrast, clear evidence for starting ART early at higher CD4^+^ T cell counts is more ambiguous and recommendations differ 226,227,228. Plausible arguments in favor of starting ART early include the likelihood of long-term clinical benefit, reduced risk of sexual HIV transmission and HSV-2 infection, concurrent treatment of HBV infection, improved tolerability of ART, reducing the latent reservoir size, and minimizing HIV-induced immune system damage 227. Potential arguments against starting ART at higher CD4^+^ counts are the absence of evidence for clinical benefit to the individual, the potential for harm from ART and the risk of inducing HIV drug resistance resulting in the lifetime limiting of treatment options 228. However, to date two studies — the TEMPRANO ANRS 12136 study 229 and the Strategic Timing of Antiretroviral Treatment (START) study 230 — provide important additional evidence to support early ART initiation by demonstrating its clinical benefits in asymptomatic patients at an early stage, when CD4^+^ cell counts are above 500 cells/mm^3^. The TEMPRANO study, involving 2056 patients in Ivory Coast, showed that early ART initiation (CD4^+^ cell count of ≥500 cells/mm^3^) was associated with a 44% lower risk of death or severe HIV-related illness than was ART initiated according to prevailing WHO criteria 231. The START study, involving 4685 patients at 215 sites in 35 countries, showed that the risk of death, a serious AIDS-related event, or a serious non-AIDS-related event was 57% lower among those treated early than among those treated when the CD4^+^ cell count decreased to 350 cells/mm^3^ 231. Patients initiating ART early in the START and TEMPRANO trials had viral suppression rates exceeding 95% and 80%, respectively. In both trials, a reduced rate of TB after early ART, as compared with deferred ART, was one of the most important contributors to the overall benefits 231. The main barrier to starting early ART is late diagnosis 232. For example, the majority of people in whom HIV is diagnosed each year in the UK have a CD4^+^ count lower than 350 cells/mm^3^ at time of testing, thus strategies are required to reduce late diagnosis 233. Currently, more than 25 single or combination HIV drugs that block HIV replication at many steps in the virus lifecycle are approved by the U.S. Food and Drug Administration (FDA) for treatment of HIV infection. The drugs are grouped into six inhibitor classes: 1) Non-nucleoside reverse transcriptase inhibitors, 2) Nucleoside reverse transcriptase inhibitors, 3) Protease inhibitors, 4) Fusion inhibitors, 5) Entry inhibitors, and 6) Integrase inhibitors. Recommended ART regimens are less toxic, more effective, have a lower pill burden, and are dosed less frequently than the initial protease inhibitor-based regimens 234.

Therapy-naïve patients in high-income countries usually start a standard ART regimen consisting of two nucleoside reverse transcriptase inhibitors (NRTIs) and either a ritonavir-boosted protease inhibitor, a non-nucleoside reverse transcriptase inhibitor (NNRTI) or an integrase inhibitor. For low-income and middle-income countries, WHO recommends a public health approach using ART with standardized first-line (NNRTI plus dual NRTIs) and second-line (ritonavir-boosted protease inhibitor plus dual NRTIs) regimens, and restricted monitoring for both efficacy and toxic effects 235. Similar virological outcomes have been reported in a study that compared a public health approach with individualized approaches to ART in a high-income versus a low-income country. However, the switching rate for toxic effects in the high-income country was higher than in the low-income country 236. After initiation of antiretroviral therapy, the plasma viral load decreases to concentrations below the lower limit of detection (< 50 copies/mL) in most people, usually within 3 months 237. By contrast, the recovery of CD4 T cells in individuals on antiretroviral therapy is variable 238. In one study the responses to ART at 6 months in low-income countries, showed that 56% of patients had a successful virological and CD4 response, 19% a virological response without a CD4 response, and 15% a CD4 response without a virological response. Individuals with impaired CD4 T-cell recovery despite virological suppression, which is associated with several risk factors, are at increased risk of adverse outcomes, including serious non-AIDS events 239,240. Early mortality rates after initiation of antiretroviral therapy are much higher in resource-limited settings than in high-income countries, however with increasing duration on ART, mortality in HIV-infected patients on treatment in a middle-income locale declines rapidly to levels approaching those in high-income settings 241. Successfully treated HIV-positive individuals have a near-normal life expectancy (other than people who inject drugs). Additionally, patients who achieve a normal CD4^+^ cell count and undetectable viral load on ART can significantly improve their life expectancy 242. Antiretroviral therapy taken in the presence of continuing viral replication will result in the selection of sub-populations of HIV with mutations conferring virologic failure and drug resistance. Sub-optimum adherence is the major factor associated with the development of resistance 243. Antiretroviral drugs differ in their ability to select for resistant mutations. Many factors determine the relative rate of resistance selection with different drugs and drug combinations. This is reflected in the "genetic barrier" to resistance, which refers to the number of mutations that must occur within a given target in order for resistance to be present against a particular drug 244. Interactions between mutations, the effects of individual resistance mutations on viral replication capacity, and viral fitness all influence mutational pathways and the overall impact of resistance mutations on viral phenotype 244. Several different mechanisms through which HIV-1 escapes from drug pressure have been described; these mechanisms differ from one drug class to another and can even differ between drugs of the same class. Also the number of mutations required for resistance to occur varies from drug to drug 244. For instance, some drugs like the NNRTIs efavirenz and nevirapine or the integrase inhibitor raltegravir as well as the combination medicines emtricitabine and lamivudine rapidly select for one mutation conferring high-level resistance, whereas most other antiretrovirals select for resistance mutations slowly and need several resistant mutations before loss of drug efficacy 99. Transmission of drug resistant virus strains is an emerging phenomenon with important clinical and public health implications. Prevalence estimates of transmission of drug-resistant HIV were found highest in North America (12.9%), followed by Europe (10.9%), Latin America (6.3%, Africa (4.7%), and Asia (4.2%) 245. Transmissions of NRTI and NNRTI resistant viruses are the most common 244. Remarkably, some HIV subtypes have a higher propensity to develop certain drug resistant mutations compared with others. For example, individuals infected with clade C have a higher incidence (70-87%) of nevirapine resistance (K103N, Y181C) compared to individuals with subtype A (42%). In addition, several studies found higher rates of the K65R mutation in clade C infected individuals treated with NRTIs compared to clade B infected individuals 246. ART resistance selection studies revealed that the K65R mutation accumulated faster under TFV pressure compared to subtype B 247. However, K65R is less frequent in subtype A than in all other subtypes 248. Further selection studies have shown that a V106M mutation commonly develops in subtype C viruses following drug pressure with nevirapine or efavirenz, unlike the V106A mutation that is more commonly selected in subtype B. The clinical relevance of this mutation has been confirmed in recent years with several studies showing that V106M is frequently seen in non-B subtypes (i.e subtype C and CRF01_AE) after therapy with efavirenz or nevirapine 249. In general, the effect of HIV-1 subtype diversity has not limited the overall benefit of ART, however, there are subtype differences in the type and preference of pathways of resistance with some mutations emerging almost exclusively in some non-B subtypes 246.

Although ART inhibits HIV replication and prevents disease progression, it does not eliminate the virus completely from infected patients, predominantly because of the presence of latently infected resting memory CD4^+^ T cells 250. These latently infected cells contain viral DNA within their chromosomes but usually express little or no viral RNA and no viral proteins, thus rendering them beyond the reach of ART and essentially invisible to the immune system. However, upon stimulation these cells can produce infectious virus and rekindle virus replication if ART is discontinued 250. In a recent study HIV sequences from resting CD4^+^ T cells from patients that were treated with ART during the acute phase of infection (within 3 months of HIV infection) were compared with those obtained from patients who initiated therapy during the chronic phase of infection. The analysis of the data revealed that known CTL escape variants were rare in acute phase-treated patients, whereas nearly all of the sequences from patients treated during the chronic phase harbored CTL escape mutations 251. Defective HIV genomes tend to accumulate in CD4^+^ cells over the course of infection, indicating that most HIV DNA present in resting CD4^+^ T cells is defective rather than latent. Interestingly, the authors also demonstrated that in contrast to what is seen in individuals treated early in infection, replication-competent HIV induced from latently infected cells from patients treated in the chronic phase also bear a large number of CTL resistance mutations 251. These data suggest that unless ART is initiated very early in the course of infection, the latent reservoir becomes populated almost exclusively with variants resistant to dominant CTL responses. Further, efforts directed toward stimulating a broader CTL response might be necessary to kill cells induced to express latent virus as well as therapeutic interventions such as, genetically engineered CTLs that are pre-programmed with T cell receptors specific for these alternative HIV epitopes, anti-HIV envelope immunotoxins, or broadly neutralizing antibodies that might also be very effective in this regard 250.

The fact that new CTL-resistant viruses largely replace the wild-type virus in the latent reservoir is of great interest as it indicates a more adaptable role of the latent reservoir, at least in the early stages of infection. This implies that the reservoir could be substantially depleted if the natural rate of elimination of latently infected cells in untreated infection could be maintained while preventing the formation anew of latency with ART, which is essentially the goal of activation-elimination approaches 250. Indeed, several methods for purging the latent reservoir have been discussed. One strategy that is being actively investigated is an activation-elimination approach in which the host cell is induced to express viral proteins, allowing it to be killed by viral cytopathic effects or by the host immune response 252. Various exogenous stimuli, including suberoylanilide hydroxamic acid, are currently being studied in efforts to safely and effectively activate latent HIV. However, some of these stimuli induce only low levels of virus expression, which might not be sufficient to kill the infected cell without a robust and effective immune response or other therapeutic intervention 253,254. Other strategies (reviewed in 255) for a functional cure besides enhancing specific immunity include 1) full or partial replacement of the immune system through genetic modifications, 2) shock and kill and 3) render HIV permanently silent. For example, the outcome of the "Berlin patient" who underwent an allogeneic stem transplant from a donor who was homozygous for the CCR5Δ32 deletion has been widely reported. In addition, Gene therapy with the aim to reduce CCR5 expression on T-cells, thus rendering them more resistant to HIV infection is currently being explored 255.

## MOLECULAR MECHANISM OF INFECTION

**Figure 6:**
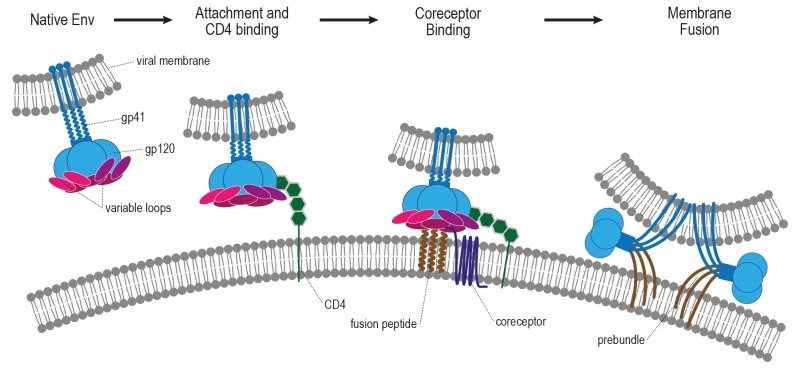
FIGURE 6: Working model of HIV-1 entry. HIV entry is initiated by attachment of gp120 to CD4, which induces a conformational change in gp120. Following engagement of coreceptor, gp120 undergoes further conformational changes that allow for the insertion of the gp41 fusion peptide into the host membrane. The formation of the six-helix bundle brings the host and viral membranes into close proximity and creates a fusion pore, allowing entry of the HIV capsid into the host cell. Adapted from 258.

The initial phase of the viral replication cycle begins with the adhesion of virus to the host cell and ends with the fusion of the cell and viral membranes with subsequent delivery of the viral core into the cytoplasm 256. The complex series of protein-protein interactions that ultimately results in virus infection can be divided into several phases (Figure 6): First, virions must bind to the target cell, by either the viral envelope (Env) protein or through host cell membrane proteins incorporated into the virion 257. Attachment can be either relatively nonspecific (e.g. Env interacting with sugar groups or lectin-like domains present on cell-surface receptors such as heparan sulfate or galactosylceramide) or more specific (e.g. interactions between Env and α4β7 integrin -the gut-homing receptor- or pattern recognition receptors such as DC-specific intercellular adhesion molecular 3-grabbing non-integrin (DC-SIGN)) 257. HIV attachment to the host cell via any of these factors likely brings Env into close proximity with the host receptor CD4 and subsequently one of the coreceptors, thus increasing the efficiency of infection. However, attachment factors are not essential, and although they enhance infection *in vitro*, their physiological role *in vivo* remains unclear 256.

The second step of virus entry and absolutely required for infection involves engagement of Env with its primary host receptor, CD4 259, which is a member of the immunoglobulin superfamily that normally functions to enhance T-cell receptor mediated signaling. Env is a heavily glyco-sylated trimer of gp120 and gp41 heterodimers and it is the sole target for HIV broadly neutralizing antibodies 260,261. The host derived N-linked glycans of the HIV Env are crucial for correct protein folding as well as viral infectivity and modulating interactions with the host immune system 262. The gp120 glycoprotein subunit is responsible for receptor binding 263 and is composed of five variable loops (V1-V5), named for their relative genetic heterogeneity, and five relatively conserved domains (C1-C5) 264. The first four variable regions form surface-exposed loops composed by disulfide bonds at their bases, with the exception of V5 265. The variable surface exposed loops on gp120 play critical roles in immune evasion and coreceptor binding. Especially, the V3 loop is the principal determinant of chemokine receptor specificity 266. Binding to the host cell CD4 receptor is mediated through the CD4 binding site (CD4bs) on gp120 and causes rearrangements of V1/V2 and subsequently V3. Additionally, CD4 binding leads to formation of the bridging sheet, a four- β strand structure comprised of two double-stranded β sheets that are spatially separated in the unliganded state 256.

The bridging sheet and the repositioned V3 loop are critical elements for coreceptor binding in the next step of virus entry 267. Coreceptor binding is widely thought to be the final trigger that activates membrane fusion 268. The relevance of viral coreceptors for subsequent HIV in-fection *in vivo* was demonstrated by the identification of a 32-base-pair deletion in CCR5, termed CCR5-Δ32, which is characterized by a premature stop codon in the second extracellular loop of CCR5 and subsequent retention of the mutant protein in the endoplasmic reticulum. The frequency of the CCR5-Δ32 allele in European Caucasians is around 10%, whereas it is absent in Africans and East Asians 269. Individuals who are CCR5-Δ32 homozygous or CCR5-Δ32/Δ32 have non-functional CCR5 receptors, resulting in profound resistance to HIV infection. However, individuals with homozygosity for CCR5-Δ32 are very rare (about 1% of Caucasians). In contrast, heterozygous individuals, who possess one copy of CCR5-Δ32 and one copy of CCR5-wildtype, are more frequent (20%) and have altered chemokine receptor activity. There is strong evidence that heterozygosity for CCR5-Δ32 provides partial protection against sexual transmission of HIV infection both from male-to-male as well as from male-to-female 269. HIV strains that use the chemokine receptor CCR5 are called R5 HIV, those that use CXCR4 are termed X4 HIV, and viruses that can use both coreceptors are called R5X4 HIV 270. Although both R5 and X4 HIV-1 variants are present in body fluids (semen, blood, cervicovaginal and rectal secretions) and despite high levels of CXCR4 expression on circulating HIV target cells, only R5 viruses are transmitted between individuals and dominate early stages of HIV disease 271.

A fourth step of virus entry involves trafficking to specific entry sites where viruses encounter a milieu that provides for productive entry and membrane fusion occurs 272. A series of studies 272,273,274 have shown that a number of viruses hijack cellular transport pathways to reach specific destinations that are either needed for infection or that make entry more efficient, and that HIV might likewise use the host cell machinery to reach sites where membrane fusion can occur 256. Some viruses, including HIV, have been shown to attach to the plasma membrane and "surf" along the cell surface, moving from distal sites of attachment to more proximal regions of the cell body where virus entry occurs 256,272,275. A recent study proposed that complete HIV fusion occurs in endosomes, as viral fusion with the plasma membrane does not progress beyond the lipid-mixing step. It was further shown that HIV virions underwent receptor-mediated internalization long before endosomal fusion, thus minimizing the surface exposure of conserved viral epitopes during fusion and reducing the efficacy of inhibitors targeting these epitopes 276.

The final step of virus entry is membrane fusion mediated by the engagement of Env with the CD4 receptor and coreceptor (i.e. CXCR4 or CCR5). Coreceptor binding induces a conformational change in Env, which causes the fusion peptide (FP) of gp41 to insert into the host cell membrane 277. Simultaneously, a coiled-coil forms comprising three adjacent amino-terminal helical regions (NHR) of gp41, the grooves of which form high affinity binding sites for the carboxy-terminal helical region (CHR) to bind in an antiparallel orientation. The result of the NHR-CHR interaction is an energetically stable 6-helix bundle (6HB), that pulls together the apposing membranes of the host cell and virus to consummate the fusion reaction 277, and results in the formation of a fusion pore 278. However, it is likely that several Env trimers are needed to form a fusion pore 268. In summary, coreceptor binding unlocks the potential energy of the gp41 fusion complex resulting in 6HB formation, opening, and stabilization of the membrane fusion pore, and subsequent delivery of the viral contents into the host cell cytoplasm.

HIV can disseminate between CD4^+^ T cells either via cell-free diffusion-limited viral spread, or by directed cell-cell transfer using virally induced structures termed virological synapses (i.e. organized contact areas, which concentrate cellular entry receptors and virions) 279. *In vitro*, HIV spreads very efficiently, if not preferentially, by cell-cell contacts from infected to non-infected cells via 1) formation of virological synapses, 2) transient cell-cell contacts, and 3) longer-range intercellular interactions including nanotubes and filopodia 280,281. Virus transmission through these mechanisms has been proven to be more efficient and rapid than infection by cell free viruses 282,283 thus supporting the notion that cell-cell transmission might be a relevant if not dominant mode of virus transmission *in vivo* 280.

Advances in electron microscopy have enabled three-dimensional-structural studies of the virological synapse that have shed light on this mechanism of infection 284. DCs, which are professional antigen presenting cells often found scavenging the periphery, produce membranous protrusions capable of trapping virions in a surface-accessible but protected compartment 285. Each DC can bind up to several hundred virions 286 most likely via a C-type lectin such as DC-SIGN 285,287. It remains unclear if these protrusions occur before or after virion binding and whether it is Env induced 256. When CD4^+^ T cells contact DCs, they extend filopodia, enriched for CD4 and coreceptor, into the invaginated DC compartments that containing bound virions. Together, the efficient binding of HIV, relocalization to the point of CD4^+^ T-cell contact, and the recruitment of the requisite HIV entry receptors promote HIV entrance at the infectious synapse 286,288. However, so far the relative contribution of cell-cell and cell-free virus transmission in acquisition of HIV infection and viral dissemination during human infection remains undefined.

In conclusion, defeating the HIV/AIDS pandemic has proven a challenging task. Nonetheless, significant advances in our understanding of the virus and the disease it causes have been transformed into improvements in the life expectancy for those affected. While a preventive vaccine or a cure has not been achieved to date, other approaches such as on-demand-PrEP appear to reduce the rate of transmission when the individuals involved adhere to a program. Our current knowledge of the virus’ biology has provided us with glimmers of solutions, and the risks derived from the improper application of therapies. Likewise, the importance of education and socio-economic factors in this endeavor cannot be overstated. Our survey of the literature shows that the continuous cooperation among all parties in the struggle against the HIV/AIDS pandemic has been vital in the advances made to date.
